# The endocannabinoid signaling system in acute and chronic pancreatitis

**DOI:** 10.3389/fphys.2026.1952582

**Published:** 2026-09-28

**Authors:** Alex Mabou Tagne, Jene Jamarillo, Lance R. McMahon, Daniele Piomelli

**Affiliations:** 1 Department of Pharmaceutical Sciences, Jerry H. Hodge School of Pharmacy, Texas Tech University Health Sciences Center, Amarillo, TX, United States; 2 Department of Anatomy and Neurobiology, University of California, Irvine, Irvine, CA, United States; 3 Department of Biological Chemistry, University of California, Irvine, Irvine, CA, United States; 4 Department of Pharmaceutical Sciences, University of California, Irvine, Irvine, CA, United States

**Keywords:** 2-arachidonoyl-sn-glycerol, acute pancreatitis, anandamide, cannabinoid, cannabis, chronic pancreatitis

## Abstract

The endocannabinoid system (ECS) is a ubiquitous lipid signaling network that regulates tissue homeostasis. Increasing evidence indicates that the ECS undergoes profound remodeling during pancreatic injury and may influence key pathological processes underlying acute pancreatitis (AP) and chronic pancreatitis (CP). Interest in this field has grown in parallel with increasing cannabis use among individuals with pancreatic disorders and the urgent need for alternatives to opioid-based pain management. Components of the ECS, including cannabinoid receptors, the endogenous ligands, and enzymes that mobilize such ligands, are widely expressed throughout both the exocrine and endocrine pancreas, where they contribute to the regulation of pancreatic function under physiological and pathological conditions. Studies demonstrate that AP and CP are associated with distinct alterations in cannabinoid receptor expression and endocannabinoid levels, yet the functional significance of these changes remains incompletely understood. Clinical evidence, however, remains limited and often conflicting. In this narrative review, we summarize current knowledge of ECS organization and function in the pancreas, examine how endocannabinoid signaling is altered during pancreatitis, and critically evaluate the therapeutic opportunities and challenges associated with targeting ECS pathways. We discuss an emerging hypothesis in which adaptive endocannabinoid signaling may contribute to resilience following pancreatic injury and propose that progressive disruption of this response could contribute to the transition from AP to CP.

## 1 Introduction

Cannabis use is growing among individuals with acute and chronic pancreatitis, where difficult-to-manage abdominal pain has led many patients to seek alternatives to conventional analgesics, including opioids (Javidi and Gardner, 2026). The analgesic properties of cannabis are primarily attributed to Δ^9^-tetrahydrocannabinol (Δ^9^-THC), a phytochemical that binds to CB_1_ and CB_2_ cannabinoid receptors to elicit many of the effects of cannabis including antinociception (Piomelli, 2005; Starowicz and Finn, 2017). CB_1_ receptors mediate most of the central and peripheral actions of Δ^9^-THC, while CB_2_ receptors mediate some of the other less-well understood effects such as those exerted on the immune system (Piomelli, 2005; Piomelli and Mabou Tagne, 2022). In contrast to exogenous cannabinoids such as Δ^9^-THC which have long half-lives and produce widespread activation of CB_1_ and CB_2_ receptors, endogenous cannabinoid signaling is tightly regulated in space and time by a network of lipid messengers, enzymes, and transport mechanisms (Di Marzo et al., 1994; Gaoni and Mechoulam, 1964). The best-studied endocannabinoids, anandamide (AEA) and 2-arachidonoyl-*sn*-glycerol (2-AG), are generated on demand and act only where and when receptor activation is needed (Bahr et al., 2006; Di Marzo et al., 1994; Gaoni and Mechoulam, 1964). Their production is mediated primarily by N-acyl phosphatidylethanolamine phospholipase D (NAPE-PLD) and diacylglycerol lipase (DGL), while their actions are terminated through cellular uptake and enzymatic degradation by fatty acid amide hydrolase (FAAH) and monoacylglycerol lipase (MGL), respectively (Di Marzo et al., 1994; Kano et al., 2009; Munro et al., 1993). Collectively, this set of molecules constitute the endocannabinoid system (ECS), a ubiquitous lipid signaling network that serves key modulatory functions in mammalian physiology (**Figure 1**).

**Figure 1:**
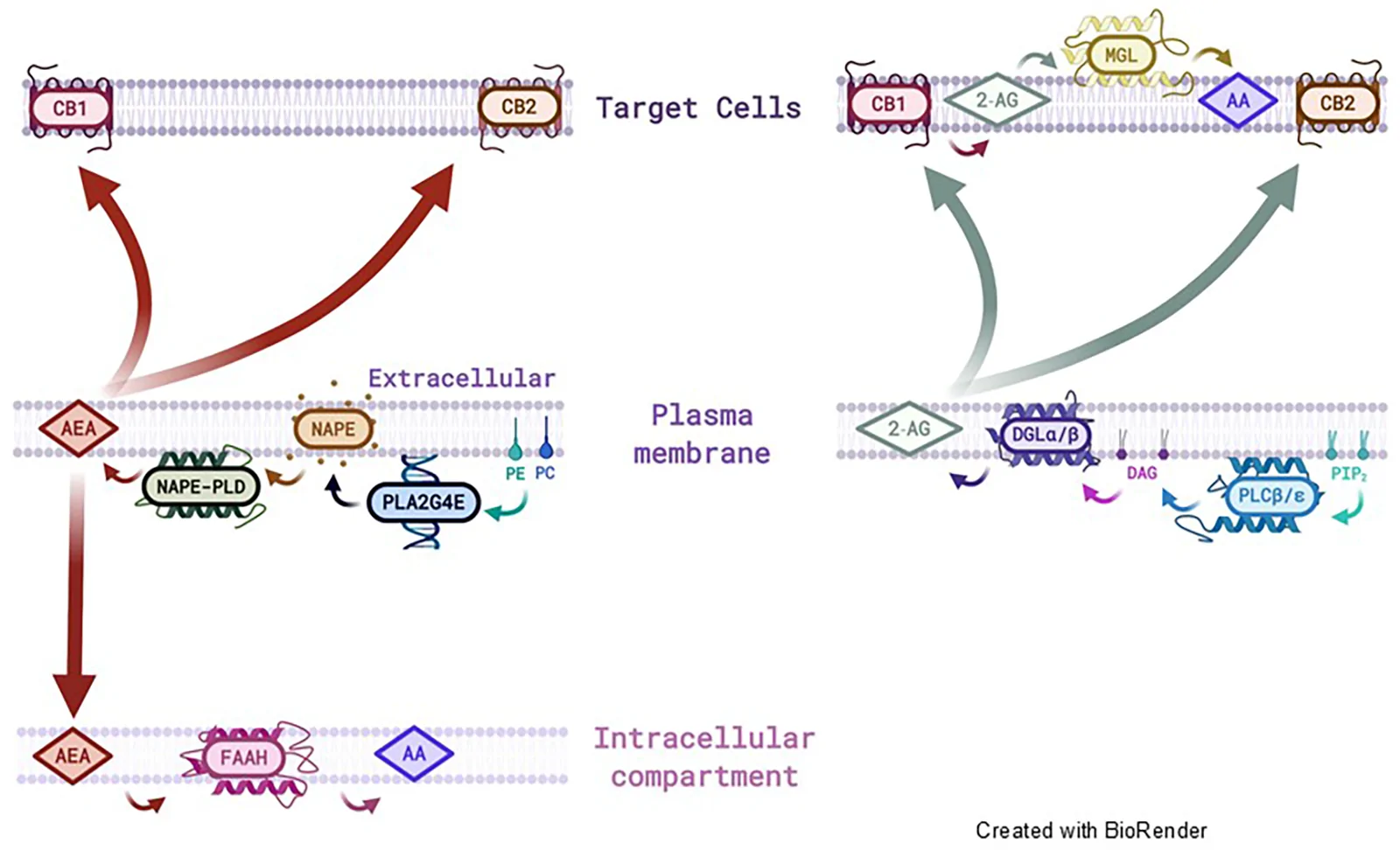
Molecular components of the endocannabinoid system. (*Left*) Anandamide (AEA) synthesis is initiated by the transfer of an arachidonoyl (AA) group from the *sn*-1 position of phosphatidylcholine (PC) to the free amino group of phosphatidylethanolamine (PE), a reaction catalyzed by calcium-dependent cytosolic phospholipase A2ϵ (PLA2G4E), generating N-arachidonoyl-phosphatidylethanolamine (NAPE). Subsequent hydrolysis of NAPE by N-acyl phosphatidylethanolamine-specific phospholipase D (NAPE-PLD) yields AEA. Following its release, AEA activates cannabinoid receptors (CB_1_ and CB_2_) on neighboring cells. Signal termination occurs primarily through intracellular hydrolysis by the serine amidase fatty acid amide hydrolase (FAAH). (*Right*) Activation of receptor-coupled phospholipase C isoforms β (PLCβ) or ϵ (PLCϵ) catalyzes the hydrolysis of phosphatidylinositol-4,5-bisphosphate (PIP_2_) to generate 1-acyl-2-arachidonoyl-*sn*-glycerol (diacylglycerol; DAG). DAG is subsequently converted to 2-arachidonoyl-*sn*-glycerol (2-AG) by diacylglycerol lipase α (DAGLα) or β (DAGLβ). 2-AG also exerts its biological effects by engaging cannabinoid receptors and is inactivated primarily by hydrolysis mediated by monoglyceride lipase (MGL).

Early investigations of the ECS were largely centered on the central nervous system, but it is now established that this lipid signaling system serves important functions in peripheral tissues (Starowicz et al., 2008). The pancreas is a notable example, as ECS components are broadly expressed throughout both exocrine and endocrine compartments (Michalski et al., 2007). Cannabinoid receptors, their endogenous ligands, as well as endocannabinoid-producing and degrading enzymes have been identified in acinar cells, ductal cells, pancreatic islets, and resident immune cells (Michalski et al., 2007, 2008). This widespread distribution positions the ECS as a critical checkpoint of pancreatic homeostasis under both physiological and pathological conditions.

Interest in pancreatic ECS signaling has intensified following the discovery that acute and chronic pancreatitis are associated with profound alterations in cannabinoid receptor expression, endocannabinoid levels, and endocannabinoid metabolic pathways (Cao et al., 2012). These changes suggest that the ECS participates in the pancreatic response to injury. However, defining the precise role of ECS has proven challenging. Emerging studies have produced seemingly contradictory findings, with ECS activation exhibiting both protective and detrimental effects depending on the experimental model, disease severity, stage of injury, cell type involved, and signaling pathway engaged. In some settings, activation of cannabinoid signaling attenuates key pathological features of pancreatitis, including oxidative stress, immune-cell recruitment, and fibrosis, while also reducing pancreatic pain (Dembiński et al., 2008; Michalski et al., 2007). In other studies, cannabinoid receptor activation has been linked to worsening pancreatic injury, metabolic dysfunction, or maladaptive inflammatory responses (Dembiński et al., 2006; Matsuda et al., 2005; Simons-Linares et al., 2019). These divergent observations have generated considerable uncertainty regarding whether ECS activation should be viewed as beneficial, harmful, or both in pancreatitis. This dual nature of ECS signaling likely reflects the remarkable complexity of the system itself (Lu and Mackie, 2021; Maccarrone et al., 2023). Broad activation or inhibition of the ECS may produce markedly different outcomes from selective modulation of specific pathways (Lu and Mackie, 2021; Maccarrone et al., 2023). Understanding these nuances is essential for interpreting the effects of cannabis use in patients with pancreatic disease and for evaluating the therapeutic potential of ECS-targeted interventions. In this review, we examine the role of endocannabinoid signaling in pancreatic physiology and pathophysiology, with particular emphasis on acute and chronic pancreatitis. We first discuss the molecular architecture and physiological functions of the ECS within the pancreas. We then review current evidence describing how endocannabinoid signaling is altered during pancreatic injury. Lastly, we critically evaluate the therapeutic opportunities and challenges associated with targeting ECS pathways in pancreatitis, highlighting both the promise and pitfalls of cannabinoid-based approaches.

## 2 Endocannabinoid signaling in normal pancreatic tissue

### 2.1 Exocrine pancreas

Within the exocrine pancreas, cannabinoid receptors are expressed in acinar cells, ductal epithelial cells, pancreatic stellate cells (PSCs), resident immune cells, and sensory nerve endings (Michalski et al., 2007, 2008) (**Figure 2**; **Tables 1**, **2**). CB_1_ receptors are particularly abundant in acinar cells, ductal cells, PSCs, and intrapancreatic neural structures, whereas CB_2_ receptors are prominently expressed by PSCs and immune cells. Both receptor subtypes have also been identified in vascular structures (Michalski et al., 2007).

**Figure 2:**
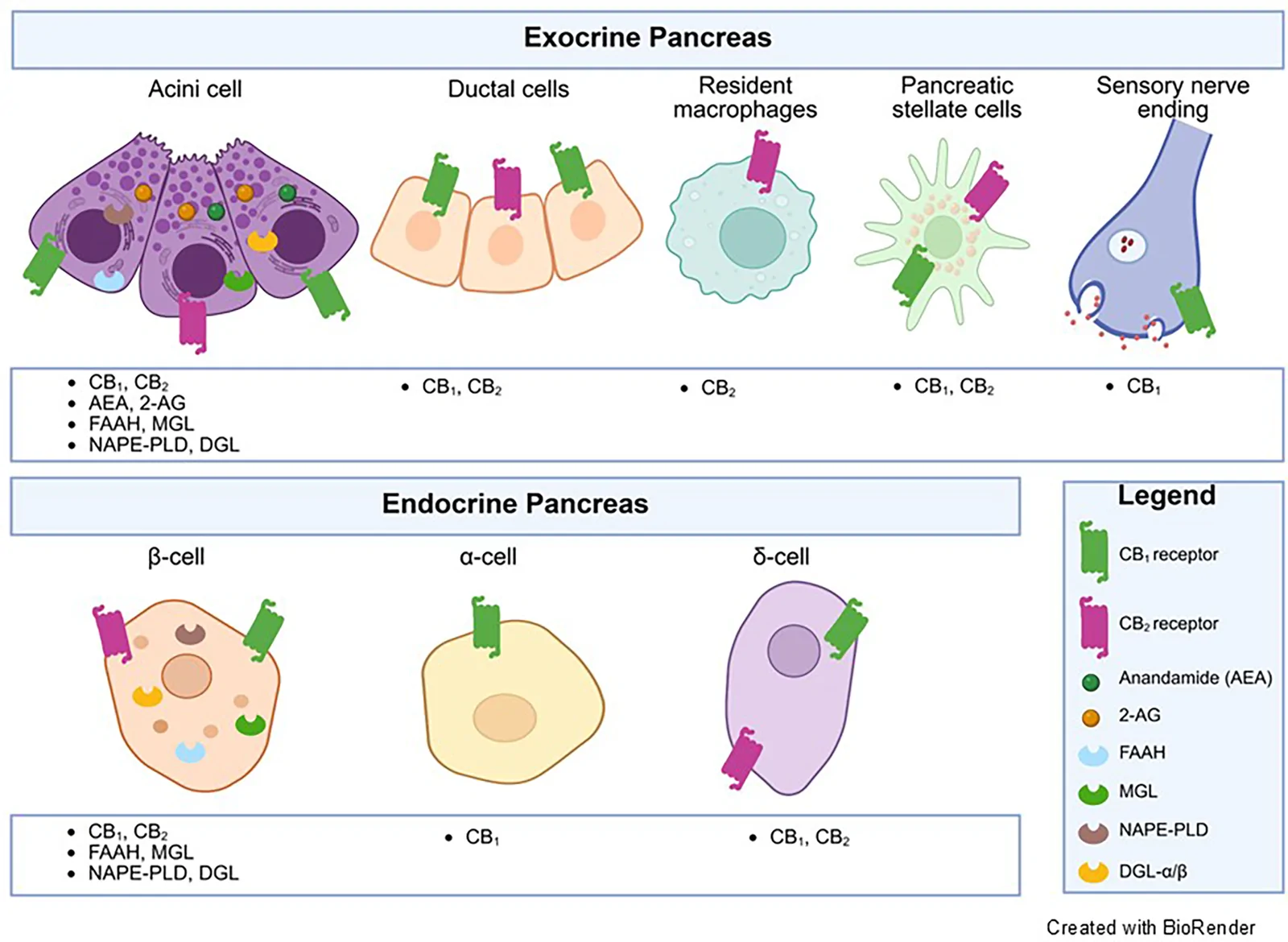
Schematic illustration of the cellular localization of cannabinoid receptors, endocannabinoids, and their metabolic enzymes in the exocrine and endocrine pancreas. Top: In the exocrine pancreas, CB_1_ and CB_2_ receptors are expressed in acinar cells, ductal cells, and pancreatic stellate cells, whereas CB_2_ receptors are predominantly associated with resident macrophages and CB_1_ receptors with sensory nerve endings. Acinar cells contain anandamide (AEA) and 2-arachidonoyl-*sn*-glycerol (2-AG), together with the major biosynthetic enzymes N-acyl phosphatidylethanolamine-specific phospholipase D (NAPE-PLD) and diacylglycerol lipase (DGL), and the degradative enzymes fatty acid amide hydrolase (FAAH) and monoglyceride lipase (MGL). Bottom: In the endocrine pancreas β-cells express CB_1_ and CB_2_ receptors together with NAPE-PLD, DGL, FAAH, and MGL, whereas α-cells predominantly express CB_1_ receptors and δ-cells express both CB_1_ and CB_2_ receptors.

**Table 1:** Distribution of endocannabinoid system components in the pancreas under physiological conditions.

ECS Component	Distribution	Species	Evidence	Reference
CB_1_ receptor	Acinar cells, ductal cells, pancreatic nerves, blood vessels, pancreatic stellate cells (PSCs), immune infiltrates	Human	Immunohistochemistry (IHC), immunofluorescence (IF), Western blot, RT-PCR	Michalski et al., 2007; Michalski et al., 2008
	α-, β-, and δ-cells	Human	IHC, IF, Western blot	Bermúdez-Silva et al., 2008
	Acinar cells, ductal cells	Mouse	IHC, Western blot, RT-PCR	Michalski et al., 2007; Michler et al., 2013
	α-, β-, and δ-cells	Mouse/Rat	IHC, IF, RT-PCR	Starowicz et al., 2008; Tharp et al., 2008; Li et al., 2010
CB_2_ receptor	Acinar cells, ductal cells, pancreatic nerves, blood vessels, PSCs, infiltrating immune cells	Human	IHC, IF, Western blot, RT-PCR	Michalski et al., 2007; Michalski et al., 2008
	δ-cells	Human	IHC, IF, Western blot	Bermúdez-Silva et al., 2008
	Acinar cells, ductal cells	Mouse	IHC, Western blot, RT-PCR	Michler et al., 2013
	β-cells	Mouse	IHC, IF, Western blot, RT-PCR	Starowicz et al., 2008; Li et al., 2010
FAAH	Pancreatic nerves, islet cells	Human	IHC, IF, Western blot, RT-PCR	Bermúdez-Silva et al., 2008; Michalski et al., 2008
	β-cells	Mouse	IHC, IF	Starowicz et al., 2008
MGL	Pancreatic nerves, islet cells	Human	IHC, IF, Western blot, RT-PCR	Bermúdez-Silva et al., 2008; Michalski et al., 2008
	α- and β-cells	Human	IHC, RT-PCR	Li et al., 2012
	β-cells	Mouse	IHC, IF	Starowicz et al., 2008; Li et al., 2012
DGL	Acinar cells, islet cells	Human	Enzymatic assay	Hou et al., 1996
	β-cells	Human/Mouse	IHC, IF, Western blot	Bermúdez-Silva et al., 2008; Starowicz et al., 2008
NAPE-PLD	Islet cells	Human	IHC, Western blot	Bermúdez-Silva et al., 2008
	β-cells	Mouse	IHC, IF	Starowicz et al., 2008
AEA (Anandamide)	Pancreatic tissue	Human	LC-MS	Michalski et al., 2007; Michalski et al., 2008
	Pancreatic tissue	Mouse	LC-MS	Starowicz et al., 2008
1-AG/2-AG	Pancreatic tissue	Human	LC-MS	Michalski et al., 2007; Michalski et al., 2008
	Pancreatic tissue	Mouse	LC-MS	Starowicz et al., 2008
PEA	β-cells	Rat	LC-APCI-MS	Matias et al., 2006
OEA	β-cells	Rat	LC-APCI-MS	Matias et al., 2006

**Table 2:** Role of endocannabinoid system components in normal pancreatic physiology.

ECS component	Function	Type of evidence	Reference
CB_1_ receptor	Activation increases insulin secretion; at low glucose enhances glucagon release and indirectly promotes somatostatin secretion.	Hormone levels (insulin, glucagon, somatostatin) measured in human islets using radioimmunoassay and Western blot	Bermúdez-Silva et al., 2008
	Activation regulates pancreatic stellate cell activity by inducing deactivation, reducing invasiveness, suppressing stress fiber formation, and decreasing proliferation and pro-inflammatory/fibrotic mediators (MMP-2, IL-6, MCP-1, fibronectin, collagen I, α-SMA).	ELISA, MTT assay, immunoblotting for protein expression	Michalski et al., 2008
	Activation in mouse β-cells decreases cAMP, increases intracellular Ca²⁺, and potentiates glucose-stimulated insulin secretion.	cAMP enzyme immunoassay, Fura-2 microfluorimetry, hormone levels (insulin, glucagon) measured in mouse islets using radioimmunoassay	Li et al., 2010
	Inhibition alters islet endocrine gene expression (↓ preproinsulin, preproglucagon, FAAH, MGL, DGL) and reduces insulin and glucagon content.	RT-qPCR and radioimmunoassay in mouse islets	Vilches-Flores et al., 2013
	Activation increases catalase activity.	Catalase activity assay (Aebi method) in rat pancreas	Coskun and Bolkent, 2014
CB_2_ receptor	Activation suppresses insulin secretion under physiological conditions.	Hormone levels (insulin, glucagon, somatostatin) measured in human islets using radioimmunoassay and Western blot	Bermúdez-Silva et al., 2008
	Activation induces quiescent PSC phenotyping, reducing inflammatory and fibrotic signaling and limiting invasiveness and proliferation.	ELISA, MTT assay, immunoblotting	Michalski et al., 2008
	Inhibition alters islet endocrine gene expression (↓ preproinsulin, preproglucagon, FAAH, MGL, DGL) and reduces insulin and glucagon content.	RT-qPCR and radioimmunoassay in mouse islets	Vilches-Flores et al., 2013
	Inhibition increases JNK phosphorylation, while activation increases p38 phosphorylation in acinar cells	Western blotting; immunohistochemistry	Michler et al., 2013
	Activation increases catalase activity, suggesting antioxidant effects in pancreatic tissue.	Catalase enzymatic assay (Aebi method)	Coskun and Bolkent, 2014
FAAH	Not defined in normal pancreatic physiology in available studies summarized here.	—	—
MGL	Inhibition increases intracellular Ca^2+^ signaling and enhances both insulin and glucagon secretion.	Hormone levels (insulin, glucagon) measured in human islets using radioimmunoassay; Fura-2 AM Ca^2+^; RT-qPCR in MIN6 cells	Li et al., 2012
DGL	DGL activity in acinar cells increases arachidonic acid mobilization and phospholipid-derived lipid signaling.	Enzymatic assays; radiolabeled lipid tracing	Hou et al., 1996
NAPE-PLD	Not defined in normal pancreatic physiology in available studies summarized here.	—	—
2-AG	Stimulates glucagon secretion under low glucose conditions.	Hormone levels (insulin, glucagon, somatostatin) measured in human islets using radioimmunoassay and Western blot	Bermúdez-Silva et al., 2008
OEA	Levels decrease in response to high glucose and insulin signaling in pancreatic β-cell models	LC-APCI-MS lipid quantification in RINm5F cells	Matias et al., 2007
AEA	Not defined in normal pancreatic physiology in available studies summarized here.	—	—
PEA	Levels decrease under high glucose/insulin stimulation.	LC-APCI-MS lipid quantification in RINm5F cells	Matias et al., 2007

Although the physiological role of cannabinoid signaling in the exocrine pancreas remains incompletely understood, available evidence suggests that the ECS contributes to cytoprotection. Activation of either CB_1_ or CB_2_ receptors increases pancreatic catalase activity and attenuates oxidative stress, indicating a role in antioxidant defense (Coskun and Bolkent, 2014). Endocannabinoid signaling has also been implicated in the regulation of PSC biology, where cannabinoid receptor activation promotes a quiescent phenotype characterized by reduced proliferation, migration, and expression of extracellular matrix proteins and inflammatory mediators (Michalski et al., 2008). These observations suggest that the ECS participates in maintaining exocrine tissue homeostasis by limiting oxidative stress and fibrogenic responses.

Both major endocannabinoids, anandamide and 2-AG, together with their biosynthetic enzymes NAPE-PLD and DGL and degradative enzymes FAAH and MGL, have been detected in pancreatic tissue (Bermúdez-Silva et al., 2008; Matias et al., 2006; Michalski et al., 2007; Starowicz et al., 2008). Although the cellular sources of these lipid mediators remain poorly defined, their anatomical distribution supports the existence of locally acting endocannabinoid signaling within the exocrine pancreas.

### 2.2 Endocrine pancreas

Cannabinoid receptor expression is not restricted to the exocrine compartment. Within pancreatic islets, Juan-Picó et al. (2006) identified both CB_1_ and CB_2_ receptors, providing early evidence for a direct role of the ECS in endocrine pancreatic function (Juan-Picó et al., 2006). CB_1_ receptors are present in α-, β-, and δ-cells of the islets of Langerhans, whereas CB_2_ receptor localization exhibits species-dependent differences, being enriched primarily in human δ-cells and rodent β-cells (Bermúdez-Silva et al., 2008; Michler et al., 2013; Starowicz et al., 2008) (**Figure 2**; **Tables 1**, **2**). Juan-Picó et al. (2006) further reported that CB_1_ receptors are mostly localized to non-β cells, whereas CB_2_ receptors are present in both β and non-β cells (Juan-Picó et al., 2006). In addition, β-cells express the enzymatic machinery required for endocannabinoid synthesis and degradation, including NAPE-PLD, DGL, FAAH, and MGL (Bermúdez-Silva et al., 2008; Matias et al., 2006; Michalski et al., 2007; Starowicz et al., 2008) (**Table 1**).

Among pancreatic cell types, β-cells have the best-characterized endocannabinoid signaling. Activation of CB_1_ receptors stimulates glucose-dependent insulin secretion, enhances intracellular Ca^2+^ signaling, and suppresses cyclic AMP concentrations (Li et al., 2010). However, CB_1_ activation may also impair insulin receptor signaling in β-cells (Kim et al., 2011), indicating that its effects on insulin secretion and insulin sensitivity are mechanistically distinct and context-dependent. In human islets, CB_1_ activation also promotes glucagon secretion and indirectly increases somatostatin release (Bermúdez-Silva et al., 2008). However, prolonged receptor activation impairs glucose responsiveness and alters endocannabinoid tone through increased FAAH expression and reduced CB_2_ receptor transcription (Vilches-Flores et al., 2013).

Endogenous 2-AG appears to be a major regulator of endocrine function. Pharmacological inhibition of MGL increases endogenous 2-AG concentrations, enhances intracellular Ca^2+^ signaling, and stimulates insulin and glucagon secretion (Li et al., 2012), while under low-glucose conditions, 2-AG preferentially promotes glucagon release (Bermúdez-Silva et al., 2008). However, Juan-Picó et al. (2006) reported that 2-AG, acting through CB_2_ receptors, modulates glucose-induced Ca^2+^ signaling in β-cells and decreases insulin secretion (Juan-Picó et al., 2006). The physiological functions of AEA within the endocrine pancreas remain less well defined.

In addition to canonical endocannabinoids, the pancreas contains structurally related lipid mediators collectively referred to as paracannabinoids, including palmitoylethanolamide (PEA) and oleoylethanolamide (OEA) (Goodman et al., 2025; Piomelli and Mabou Tagne, 2022). Although these compounds do not activate CB_1_ or CB_2_ receptors, they engage complementary signaling pathways, particularly through peroxisome proliferator-activated receptor-α (PPARα), and can influence inflammation, metabolism, and cellular stress responses (Fu et al., 2003; Lo Verme et al., 2005; Piomelli and Mabou Tagne, 2022; Piomelli et al., 2020). Emerging evidence suggests that PEA and OEA may contribute to pancreatic homeostasis and adaptation to injury (Matias et al., 2007), yet their specific roles in pancreatic disease remain largely unexplored.

## 3 Endocannabinoid signals in diseased pancreatic tissue

### 3.1 Acute pancreatitis

Acute pancreatitis (AP) is associated with marked changes in pancreatic endocannabinoid signaling (**Table 3**). Studies in both human tissue and experimental rodent models have reported increased CB_1_ and CB_2_ receptor expression in acinar cells, ductal epithelium, and intrapancreatic nerves, particularly in regions adjacent to necrotic lesions (Michalski et al., 2007). These receptor changes are accompanied by marked alterations in endocannabinoid levels. Most notably, pancreatic AEA concentrations are increased approximately 6.6-fold in patients with acute pancreatitis, whereas 2-AG levels remain largely unchanged (Michalski et al., 2007). Similarly, circulating AEA concentrations are elevated in rats with severe necrotizing pancreatitis compared with mild edematous disease and healthy controls (Matsuda et al., 2005). Although these early studies established that the ECS is profoundly altered during acute pancreatic injury, their interpretation is limited by their cross-sectional design. Human tissue samples were obtained from patients with clinically established disease, providing only a single snapshot of ECS activity without information regarding disease onset, progression, or recovery (Michalski et al., 2007). Likewise, most experimental studies examined receptor expression at isolated time points rather than across the full course of disease (Matsuda et al., 2005). Subsequent work demonstrated selective upregulation of CB_2_, but not CB_1_, in cerulein-induced pancreatitis, with CB_2_ expression enriched in apoptotic cells (Michler et al., 2013), suggesting that ECS remodeling is endocannabinoid- and cell type-specific and evolves during disease progression.

**Table 3:** Alterations in endocannabinoid signaling during acute and chronic pancreatitis.

ECS Component	Pathological Condition	Alteration in Disease	Type of Evidence	Reference
CB_1_ receptor	Acute pancreatitis	↑ Expression in acinar cells adjacent to necrotic areas in human and murine pancreatitis	IHC, Western blot, RT-PCR	Michalski et al., 2007
		↔/Slight ↑ expression compared with non-pancreatitis tissue	IHC, Western blot, RT-PCR	Michler et al., 2013
		↑ Acinar cell CB_1_ expression in cerulein-induced pancreatitis	IHC, Western blot	Cao et al., 2012
	Chronic pancreatitis	↑ Expression in inflammatory regions, pancreatic stellate cells, and nerves	IF, IHC, ICC	Michalski et al., 2008
CB_2_ receptor	Acute pancreatitis	↑ Expression in acinar cells surrounding necrotic lesions	IHC, Western blot, RT-PCR	Michalski et al., 2007
	Acute pancreatitis	Marked ↑ expression in apoptotic acinar cells	IHC, Western blot, RT-PCR	Michler et al., 2013
	Acute pancreatitis	↑ Acinar cell CB_2_ expression in experimental pancreatitis	IHC, Western blot	Cao et al., 2012
	Chronic pancreatitis	↑ Expression in inflammatory regions, stellate cells, and nerves	IF, IHC, ICC	Michalski et al., 2008
FAAH	Acute and chronic pancreatitis	Not reported	—	—
MGL	Acute pancreatitis	↑ pancreatic *Mgll* expression	RT-qPCR	Goncalves-Romeu et al., 2026
ABHD6	Acute pancreatitis	↑ pancreatic *Abhd6* expression	RT-qPCR	Goncalves-Romeu et al., 2026
DGLα	Acute and chronic pancreatitis	Not reported	—	—
NAPE-PLD	Acute and chronic pancreatitis	Not reported	—	—
1-AG/2-AG	Acute pancreatitis	↓ circulating 2-AG levels in severe human and experimental acute pancreatitis	LC-MS	Goncalves-Romeu et al., 2026
	Chronic pancreatitis	↓ 4.2-fold compared with normal pancreas	LC-MS	Michalski et al., 2008
AEA	Acute pancreatitis	↑ 6.6-fold compared with normal pancreas	LC-MS	Michalski et al., 2007
	Chronic pancreatitis	↓ Trend (2.2-fold), not statistically significant	LC-MS	Michalski et al., 2008
OEA	Acute and chronic pancreatitis	Not reported	—	—
PEA	Acute and chronic pancreatitis	Not reported	—	—

↑ Increase and ↓ Decrease.

Recent findings have substantially refined this view by revealing that ECS remodeling during AP is far more dynamic than previously appreciated. Goncalves-Romeu et al. (2026) reported that circulating 2-AG concentrations are preserved or modestly increased in mild AP but decline markedly in patients with severe disease and in mice with cerulein-induced pancreatitis (Goncalves-Romeu et al., 2026). This reduction was accompanied by increased pancreatic expression of the degradative enzymes MGL and α/β-hydrolase domain-containing protein 6 (ABHD6), indicating that enhanced 2-AG catabolism contributes to the loss of endogenous cannabinoid tone during severe disease (Goncalves-Romeu et al., 2026). Notably, these changes occurred despite relatively modest alterations in cannabinoid receptor expression (Goncalves-Romeu et al., 2026), suggesting that disease progression is driven not only by changes in receptor expression but also by profound shifts in endocannabinoid metabolism. These observations challenge the earlier concept of generalized ECS activation during AP and instead support a model in which severe disease is characterized by selective depletion of protective 2-AG signaling. Whether this loss of endogenous 2-AG contributes causally to the transition from localized pancreatic injury to systemic inflammation or represents a compensatory consequence of disease progression remains to be determined.

### 3.2 Chronic pancreatitis

In contrast to acute pancreatitis, chronic pancreatitis (CP) is characterized by a distinct pattern of changes in ECS (**Table 3**). Analyses of pancreatic tissue from patients undergoing surgery for chronic pancreatitis revealed increased CB_1_ and CB_2_ receptor expression in PSCs, intrapancreatic nerves, and infiltrating immune cells (Michalski et al., 2008). More importantly, this receptor upregulation occurred alongside reduced endocannabinoid levels, with pancreatic 2-AG concentrations decreasing approximately fourfold and AEA levels showing a similar downward trend (Michalski et al., 2008). These findings suggest a state of relative endocannabinoid deficiency despite enhanced receptor availability. The mechanisms underlying this imbalance remain poorly understood and may involve altered endocannabinoid biosynthesis, increased degradation, or reduced precursor availability. Importantly, impaired endocannabinoid signaling has been linked to PSC activation and the acquisition of a profibrotic phenotype, raising the possibility that ECS dysfunction contributes to fibrosis and disease progression (Michalski et al., 2008). However, it remains uncertain whether these alterations are causative drivers of chronic pancreatitis or secondary consequences of ongoing tissue remodeling.

### 3.3 Circulating endocannabinoids as biomarkers of pancreatic disease

Growing evidence suggests that circulating endocannabinoids may serve as accessible biomarkers of pancreatic inflammation, pain, and disease severity. A major advance came from Goodman et al. (2025), who comprehensively profiled circulating endocannabinoids and paracannabinoids across the spectrum of pancreatic disease using serum samples from the multicenter PROCEED consortium. The investigators quantified AEA, 2-AG, oleoylethanolamide (OEA), and palmitoylethanolamide (PEA) in patients with acute pancreatitis, recurrent acute pancreatitis, chronic pancreatitis, and chronic abdominal pain of suspected pancreatic origin (Goodman et al., 2025). Consistent with earlier reports of increased pancreatic AEA levels in pancreatitis, circulating AEA concentrations were also elevated across pancreatic disease states, with the highest levels observed in patients with AP, suggesting that ECS alterations are detectable not only within the pancreas but also systemically (Goodman et al., 2025). In contrast, individuals reporting abdominal pain exhibited lower circulating concentrations of 2-AG, OEA, and PEA, and pain severity was inversely correlated with circulating 2-AG and OEA levels. These findings suggest that distinct endocannabinoid mediators may differentially reflect inflammatory activity and pain processing during pancreatic disease.

More recently, Goncalves-Romeu et al. (2026) extended these observations by demonstrating that circulating 2-AG concentrations are markedly reduced in patients with severe AP compared with patients with mild disease and healthy controls, a finding that was replicated in a mouse model of cerulein-induced AP (Goncalves-Romeu et al., 2026). The decline in circulating 2-AG was accompanied by increased pancreatic expression of the degradative enzymes MGL and ABHD6, linking reduced systemic 2-AG levels to enhanced endocannabinoid catabolism rather than diminished synthesis alone (Goncalves-Romeu et al., 2026). Together, these studies indicate that circulating endocannabinoid profiles are dynamically altered during AP, with elevated AEA and reduced 2-AG associated with distinct stages and severity of the disease.

Despite these encouraging findings, several limitations warrant consideration. Both studies employed cross-sectional designs, precluding assessment of longitudinal changes in endocannabinoid signaling during disease initiation, progression, and resolution. In addition, circulating systemic endocannabinoid concentrations may not accurately reflect local pancreatic ECS activity and may be influenced by multiple biological and pre-analytical variables, including sample handling, nutritional status, and metabolic state. Consequently, whether measuring any single endocannabinoid can accurately differentiate healthy individuals from patients with disease or distinguish between disease stages remains uncertain. Future longitudinal studies integrating circulating lipidomics with pancreatic tissue analyses and clinical outcomes will be instrumental to determine whether dynamic changes in endocannabinoid profiles can improve risk stratification, predict disease progression, or guide therapeutic interventions. Nevertheless, the available evidence supports the idea that circulating endocannabinoids represent a promising class of minimally invasive biomarkers capable of providing complementary information on inflammatory burden, pain phenotypes, and disease severity throughout the course of pancreatitis.

## 4 ECS-targeted interventions for acute and chronic pancreatitis

### 4.1 Acute pancreatitis

Early investigations suggested that excessive ECS activation contributes to the pathophysiology of AP (**Table 4**). Matsuda and colleagues reported substantial elevations in circulating AEA during severe necrotizing pancreatitis in rats and demonstrated that CB_1_ receptor blockade with AM251 improved mean arterial pressure and survival (Matsuda et al., 2005). These protective effects occurred without significant modification of local pancreatic injury (Matsuda et al., 2005), indicating that CB_1_ signaling primarily contributes to the cardiocirculatory dysfunction and systemic consequences of severe AP rather than directly driving pancreatic necrosis. These findings identified endocannabinoid-mediated cardiovascular dysfunction as a potential therapeutic target in severe disease.

**Table 4:** Effects of endocannabinoid-based interventions in pancreatic diseases.

Disease	Intervention/Target pathway	ECS Target	Experimental Model	Key Findings	Reference
Acute pancreatitis	AM251 (CB_1_ receptor antagonist)	CB_1_	Cerulein- and sodium taurocholate-induced pancreatitis (rats)	Counteracts circulatory collapse; significantly improves mean arterial pressure and overall survival without directly altering local pancreatic tissue injury.	Matsuda et al., 2005
Acute pancreatitis	Exogenous anandamide (Administered prior to disease induction)	CB_1_/CB_2_	Cerulein-induced acute pancreatitis model (rats)	Exacerbates disease severity; increases pancreatic edema, inflammatory cell infiltration, pro-inflammatory cytokine production, and serum digestive enzyme levels.	Dembiński et al., 2006, 2008
Acute pancreatitis	AM251 (CB_1_ receptor antagonist) Exogenous anandamide (Administered prior to disease induction)	CB_1_	Cerulein-induced acute pancreatitis model (rats)	Exerts a local protective role; directly attenuates both histological and biochemical markers of early pancreatic injury.	Dembiński et al., 2006
Acute pancreatitis	AM251 (CB_1_ receptor antagonist) AM630 (CB_2_ receptor antagonist) HU210 (Nonselective CB_1_/CB_2_ receptor agonist in established disease)	CB_1_/CB_2_	Cerulein model of acute pancreatitis (mice/rodents)	Increases abdominal pain responses, demonstrating that endogenous cannabinoid signaling tonically suppresses pancreatitis-associated pain.	Michalski et al., 2007
Acute pancreatitis	HU210 (Nonselective CB_1_/CB_2_ receptor agonist in established disease)	CB_1_/CB_2_	Cerulein model of acute pancreatitis (mice/rodents)	Provides significant analgesia; reduces abdominal pain, pancreatic edema, inflammatory cytokine production, and established tissue injury without causing overt behavioral side effects.	Michalski et al., 2007
Acute Pancreatitis	Exogenous Anandamide (Administered after disease onset)	CB_1_/CB_2_	Cerulein-induced acute pancreatitis model (rats)	Mitigates damage; reduces pancreatic tissue injury, dampens inflammatory mediator production, decreases digestive enzyme release, and promotes active tissue recovery.	Dembiński et al., 2008
Acute pancreatitis	JZL184 (MGL inhibitor) or exogenous 2-AG	CB_1_/CB_2_	Cerulein-induced acute pancreatitis (mice)	Restores 2-AG signaling, reduces pancreatic edema, suppresses systemic inflammation and lung injury, and promotes anti-inflammatory macrophage polarization.	Goncalves-Romeu et al., 2026
Chronic pancreatitis	WIN55,212-2 (Nonselective CB_1_/CB_2_ receptor agonist)	CB_1_/CB_2_	In vitro cell culture of isolated pancreatic stellate cells (PSCs)	Acts as an endogenous brake on fibrosis; suppresses PSC activation and promotes a quiescent phenotype characterized by reduced expression of type I collagen, fibronectin, IL-6, and MCP-1 discontinuation in several patients.	Michalski et al., 2008
Chronic pancreatitis	LY3038404 (Selective CB_2_ receptor agonist)	CB_1_/CB_2_	Alcohol- and high-fat diet-induced chronic pancreatitis model (rats)	Significantly reduces visceral pain behaviors (mechanical allodynia, thermal hyperalgesia) without altering motor/cognitive function; attenuates pancreatic degeneration, fibrosis, fat deposition, and Ki67 expression.	Zhang et al., 2014
Chronic pancreatitis	Single oral dose of Δ^9^-THC (Namisol^®^)	CB_1_/CB_2_	Randomized, placebo-controlled crossover clinical trial	Fails to provide significant analgesia compared to active placebo; does not alter circulating pro-inflammatory cytokine concentrations (TNF-α, IL-8, IL-10) and increases heart rate and anxiety.	de Vries et al., 2016; Utomo et al., 2015
Chronic pancreatitis	Cannabidiol-rich cannabis extract	Not specified	Individual clinical patient case report	Produced complete pain relief, eliminated recurrent pancreatitis-related hospitalizations, improved appetite, promoted weight gain, and enhanced overall quality of life in a patient with refractory disease.	Spaccavento et al., 2025

Subsequent studies provided evidence for a direct pathogenic role of pancreatic CB_1_ signaling. Dembiński and colleagues demonstrated that exogenous AEA administration worsened cerulein-induced pancreatitis, increasing pancreatic edema, inflammatory cell infiltration, pro-inflammatory cytokine expression, and circulating digestive enzyme levels (Dembiński et al., 2006). Conversely, pharmacological CB_1_ antagonism reduced both histological and biochemical markers of pancreatic injury (Dembiński et al., 2006). The authors proposed that excessive CB_1_ activation may exacerbate pancreatic injury through inhibition of capsaicin-sensitive sensory neurons, which normally exert protective effects in the pancreas by regulating local inflammatory and vascular responses (Dembiński et al., 2003; Dembinski et al., 1996; Warzecha et al., 1997). Together, these findings established early ECS activation, particularly through CB_1_ receptors, as a potential contributor to AP progression.

However, accumulating evidence indicates that ECS activation may also exert protective effects once pancreatic injury is established (**Table 4**). Using a cerulein model of AP, Michalski and colleagues demonstrated that pharmacological blockade of either CB_1_ or CB_2_ receptors increased abdominal pain-related behaviors, suggesting that endogenous cannabinoid signaling provides tonic inhibition of pancreatitis-associated nociception (Michalski et al., 2007). Confirming this, administration of the nonselective CB_1_/CB_2_ agonist HU210 reduced abdominal pain, pancreatic edema, inflammatory cytokine production, and tissue injury without producing overt cannabinoid-related behavioral effects (Michalski et al., 2007). These observations indicate that, once inflammation is established, ECS activation may transition from a pathogenic to a compensatory mechanism by limiting inflammatory responses and suppressing pain.

A mechanistic explanation for this apparent duality emerged from studies demonstrating a biphasic, time-dependent effect of AEA in AP (Dembiński et al., 2008). When administered before pancreatitis induction, AEA exacerbated pancreatic injury and inflammation, whereas administration after disease onset reduced tissue damage, inflammatory mediator production, and digestive enzyme release while enhancing recovery (Dembiński et al., 2008). Thus, the physiological significance of ECS activation appears to depend critically on the stage of disease, with early activation exacerbating disease initiation and later ECS engagement supporting resolution and tissue repair.

Recent work has further expanded this notion by demonstrating that enhancing endogenous 2-AG signaling through inhibition of its degradation produces systemic anti-inflammatory effects during AP (Goncalves-Romeu et al., 2026). Pharmacological inhibition of MGL with JZL184 restored circulating 2-AG levels, suppressed systemic TNF-α and IL-1β production, promoted macrophage polarization from a pro-inflammatory CD80^+^ phenotype toward a reparative state, and markedly reduced AP-associated lung injury (Goncalves-Romeu et al., 2026). Interestingly, improvements in pancreatic tissue damage were comparatively modest, suggesting that 2-AG signaling primarily regulates systemic immune activation and extra-pancreatic complications rather than the initial pancreatic insult. Genetic deletion of CB_1_ receptors and pharmacological blockade of CB_2_ receptors further revealed distinct but overlapping roles for CB1 and CB2 receptors in controlling immune responses and pulmonary inflammation (Goncalves-Romeu et al., 2026), highlighting the complexity of receptor-specific ECS functions during AP. Collectively, these findings suggest that preserving endogenous 2-AG signaling through MGL inhibition may represent a promising strategy for mitigating systemic complications associated with severe AP.

Taken together, preclinical studies indicate that ECS signaling exerts both beneficial and detrimental effects during AP. Excessive early CB_1_ receptor activation contributes to pancreatic injury, inflammation, and cardiocirculatory dysfunction, whereas ECS activation during established disease may provide analgesic, immunoregulatory, and tissue-protective effects. This temporal dichotomy emphasizes that therapeutic strategies targeting the ECS will likely require precise control of receptor subtype, cellular localization, and timing of intervention rather than generalized cannabinoid receptor activation or inhibition.

The clinical relevance of these observations remains unresolved. Several case reports and systematic reviews have implicated cannabis exposure as a potential cause of AP, particularly among younger individuals and frequent cannabis users (Barkin et al., 2017; Grant and Gandhi, 2004; Lubega and Lwanga, 2021; Tanvir et al., 2024). Proposed mechanisms include CB_1_-dependent disruption of acinar cell calcium homeostasis, premature digestive enzyme activation, and alterations in pancreatic microcirculation (Tanvir et al., 2024). Importantly, recurrence of pancreatitis after continued cannabis exposure and resolution following cessation in some patients support a possible causal relationship in susceptible individuals.

Conversely, large retrospective analyses have reported associations between cannabis use and improved outcomes among patients hospitalized with AP. Studies involving large national cohorts have linked cannabis exposure with reduced inpatient mortality, shorter hospital stays, lower healthcare costs, and decreased incidence of severe complications, including shock, acute kidney injury, and respiratory failure (Simons-Linares et al., 2019). Similarly, cannabis users with alcohol-associated pancreatitis have been reported to present with lower disease severity and reduced systemic inflammatory responses (Goyal et al., 2017). Cannabis exposure has also been associated with a lower risk of AP among individuals with alcohol use disorder, although this relationship does not appear to extend to gallstone-associated disease (Adejumo et al., 2019). These conflicting clinical observations likely reflect substantial heterogeneity in cannabinoid exposure, including differences in cannabinoid composition, dose, route of administration, duration of use, patient demographics, and underlying AP etiology. Furthermore, most available clinical studies are retrospective and vulnerable to residual confounding, limiting causal interpretation. Thus, current evidence does not support a binary classification of cannabinoid signaling as either detrimental or protective in AP. AP therefore represents a paradigmatic example of the context-dependent nature of ECS biology in pancreatic disease. Depending on the molecular pathway engaged, disease stage, and cellular compartment involved, ECS signaling can contribute to inflammation and tissue injury, suppress abdominal pain, regulate immune responses, or promote recovery. These complexities highlight the limitations of broad cannabinoid receptor targeting and support the development of more selective approaches, including peripherally restricted and tissue-specific CB_1_ antagonists, CB_2_-selective agonists, inhibitors of endocannabinoid degradation, and cell-specific modulation of ECS pathways. Defining the temporal and cellular determinants of ECS activity will be essential for translating cannabinoid-based strategies into safe and effective therapies for AP.

### 4.2 Chronic pancreatitis

Evidence from human pancreatic tissue indicates that the ECS undergoes substantial remodeling during CP (**Table 4**). Although pancreatic levels of endocannabinoids, particularly 2-AG, are reduced, expression of CB_1_ and CB_2_ receptors is increased in PSCs, intrapancreatic nerves, and inflammatory cells (Michalski et al., 2008). This pattern suggests the existence of a relative endocannabinoid deficiency despite enhanced receptor availability. Importantly, activation of cannabinoid receptors with the non-selective agonist WIN55,212–2 suppresses PSC activation and promotes a quiescent phenotype characterized by reduced expression of type I collagen, fibronectin, IL-6, and MCP-1 (Michalski et al., 2008). These findings identify endocannabinoid signaling as a potential endogenous brake on pancreatic fibrosis and inflammation. Studies using receptor-selective pharmacological tools further suggest that both CB_1_ and CB_2_ receptors contribute to these antifibrotic effects (Michalski et al., 2008).

Among ECS-based interventions, selective CB_2_ receptor activation has produced particularly encouraging results in preclinical models. In a rat model of alcohol- and high-fat diet-induced CP, treatment with the selective CB_2_ agonist LY3038404 significantly reduced visceral pain-related behaviors, including mechanical allodynia and thermal hyperalgesia, while preserving normal motor and cognitive function (Zhang et al., 2014). Beyond its analgesic effects, CB_2_ activation attenuated pancreatic degeneration, fibrosis, and fat deposition and reduced expression of the proliferation marker Ki67, suggesting broad anti-inflammatory and tissue-protective actions. These findings support the notion that selective CB_2_ agonists may offer therapeutic advantages by providing analgesia without the psychoactive effects associated with CB_1_ receptor activation.

Despite these promising preclinical findings, clinical evidence remains limited and mixed. In a randomized crossover trial involving patients with CP-associated abdominal pain, a single oral dose of Δ^9^-THC (Namisol^®^) failed to produce significant analgesia compared with an active placebo (de Vries et al., 2016). Although the drug was generally well tolerated, it increased heart rate and anxiety and did not significantly improve pain scores (de Vries et al., 2016). Additionally, Δ^9^-THC did not significantly alter circulating concentrations of pro-inflammatory cytokines, including TNF-α, IL-8, and IL-10 (Utomo et al., 2015). Collectively, these studies suggest that Δ^9^-THC may have limited therapeutic efficacy in CP, although the small sample sizes (*n* = 12) and heterogeneity of patient populations warrant cautious interpretation.

In contrast, observational studies have reported more favorable outcomes associated with medical cannabis use. A retrospective cohort study of patients with CP-related pain found that enrollment in medical cannabis programs was associated with reductions in opioid consumption, hospital admissions, and emergency department visits, although several outcomes did not reach statistical significance because of limited sample size (Barlowe et al., 2019). Similarly, large database analyses have associated cannabis use with reduced mortality, lower rates of intensive care unit admission, and fewer thromboembolic complications among hospitalized individuals with CP (Sohal et al., 2025). While these findings suggest potential therapeutic benefit, they should be interpreted cautiously given the retrospective nature of the studies and the inability to account fully for confounding factors.

Case reports have also highlighted the potential therapeutic value of cannabinoid-based interventions. For example, treatment with a cannabidiol-rich cannabis extract reportedly produced complete pain relief, eliminated recurrent pancreatitis-related hospitalizations, improved appetite and body weight, and enhanced overall quality of life in a patient with refractory CP (Spaccavento et al., 2025). Although anecdotal, such observations raise the possibility that cannabidiol, a cannabis constituent that does not engage cannabinoid receptors, may provide broader therapeutic benefits than Δ^9^-THC-dominant cannabis.

Overall, the available evidence suggests that ECS modulation may represent a promising therapeutic strategy for CP. Preclinical studies consistently demonstrate anti-inflammatory, antifibrotic, and analgesic effects, particularly following CB_2_ receptor activation, whereas clinical studies have yielded more variable results. The discrepancy between experimental and clinical findings likely reflects differences in cannabinoid composition, receptor selectivity, dosing regimens, disease stage, and patient heterogeneity. Well-powered randomized clinical trials will be necessary to determine whether the encouraging preclinical effects of ECS-targeted therapies can be translated into meaningful clinical benefit for patients with chronic pancreatitis.

## 5 Discussion

The ECS has emerged as a critical checkpoint of pancreatic homeostasis and disease progression. Over the past two decades, accumulating evidence has demonstrated that cannabinoid receptors, endogenous lipid mediators, and their metabolic enzymes undergo dynamic remodeling during both acute and chronic pancreatitis. Experimental studies indicate that ECS signaling influences several pathological processes central to pancreatitis, including immune activation, inflammatory resolution, nociceptive processing, PSC activation, and fibrosis. However, fundamental aspects of ECS biology in pancreatic disease remain unresolved. The cellular sources and spatial distribution of endocannabinoids during injury, the mechanisms generating distinct ECS signatures across disease stages, and the causal contribution of these alterations to pancreatitis progression remain incompletely defined.

Importantly, the strength and nature of the evidence supporting ECS involvement in pancreatitis vary substantially across experimental systems. Human pancreatic tissue studies provide observational evidence that cannabinoid receptor expression and endocannabinoid concentrations are altered during AP and CP, but their predominantly cross-sectional design does not establish whether these changes are causal, compensatory, or secondary to tissue injury. Animal studies provide stronger mechanistic evidence, demonstrating that pharmacological or genetic manipulation of ECS pathways can modify pancreatic inflammation, nociceptive behavior, and systemic complications. *In vitro* studies, particularly those using isolated PSCs, further support direct effects of cannabinoid receptor signaling on cellular activation and fibrogenic responses, although these reductionist systems cannot reproduce the full complexity of pancreatic disease. By contrast, human observational studies of cannabis exposure are largely retrospective and vulnerable to confounding, whereas interventional clinical evidence remains limited.

An emerging hypothesis is that the ECS may serve as an endogenous adaptive system that limits excessive inflammatory and nociceptive signaling following pancreatic injury (**Figures 3**, **4**). While early investigations focused primarily on increased AEA production and cannabinoid receptor upregulation after pancreatic injury, more recent evidence has revealed that severe AP is associated with depletion of circulating 2-AG resulting from enhanced metabolic degradation (Goncalves-Romeu et al., 2026). Restoration of endogenous 2-AG signaling through MGL inhibition suppresses systemic inflammatory responses, promotes a macrophage reparative phenotype, and reduces pancreatitis-associated pulmonary complications (Goncalves-Romeu et al., 2026). Together, these observations support the possibility that changes in endocannabinoid tone may influence the balance between injury amplification and resolution, although this interpretation requires further experimental validation.

**Figure 3:**
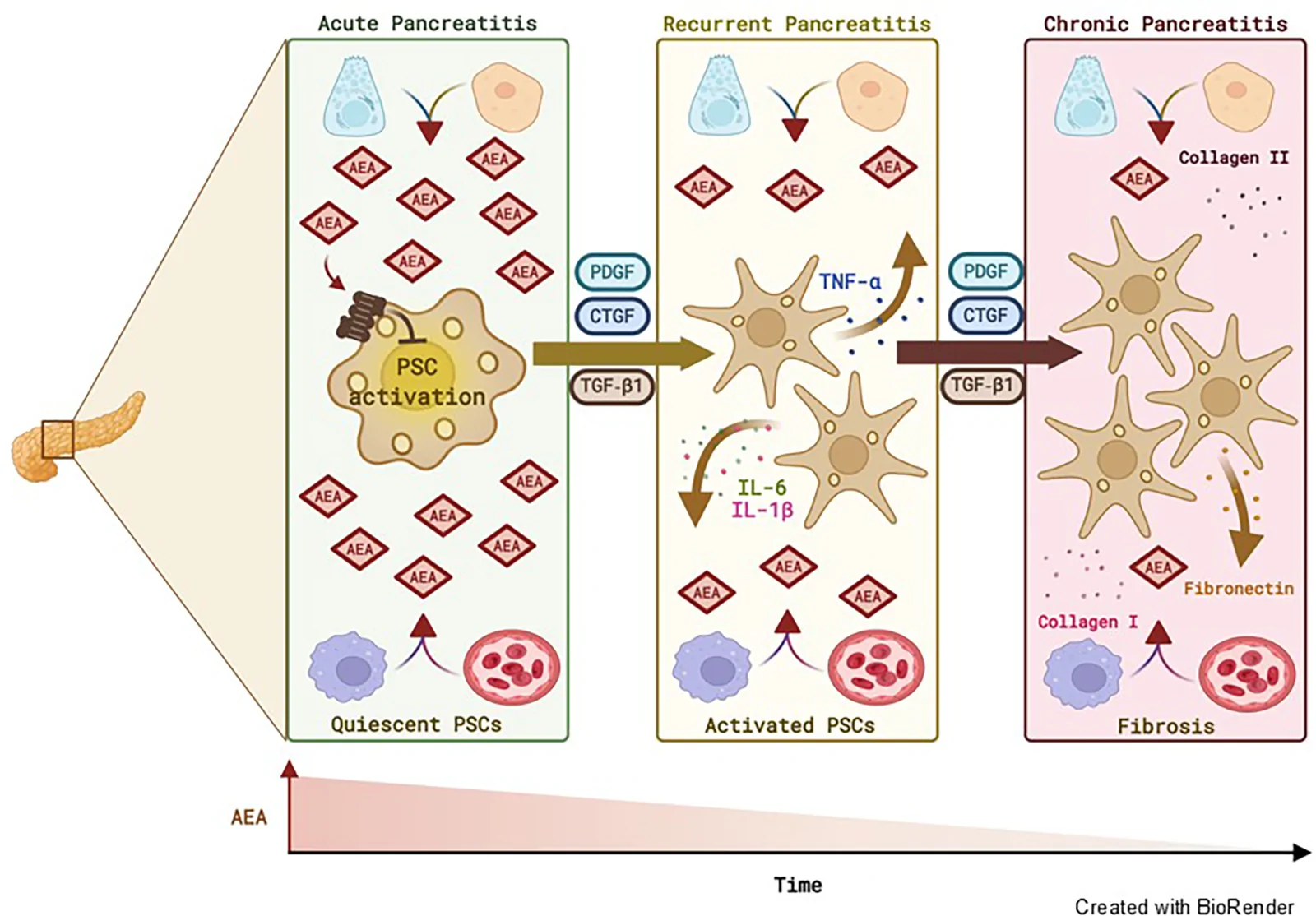
Proposed model of endocannabinoid signaling system remodeling during the transition from acute pancreatitis (AP) to chronic pancreatitis (CP). Acute pancreatic injury increases local production of the endocannabinoid anandamide (AEA) and induces the release of profibrotic mediators, including transforming growth factor-β1 (TGF-β1), platelet-derived growth factor (PDGF), and connective tissue growth factor (CTGF). Under physiological conditions, AEA signaling helps maintain pancreatic stellate cells (PSCs) in a relatively quiescent state and limits excessive extracellular matrix (ECM) deposition. Recurrent acute pancreatitis (RAP) is associated with progressive impairment of endocannabinoid signaling, resulting in reduced cannabinoid receptor-mediated restraint of PSC activation. Activated PSCs acquire a myofibroblast-like phenotype characterized by enhanced proliferation, migration, and production of ECM proteins such as collagen and fibronectin. Concurrent increases in inflammatory mediators, including interleukin-1β (IL-1β), interleukin-6 (IL-6), and tumor necrosis factor-α (TNF-α), further promote pancreatic fibrosis and tissue remodeling. Together, persistent inflammation, stellate cell activation, and reduced endocannabinoid tone contribute to the transition from RAP to CP.

**Figure 4:**
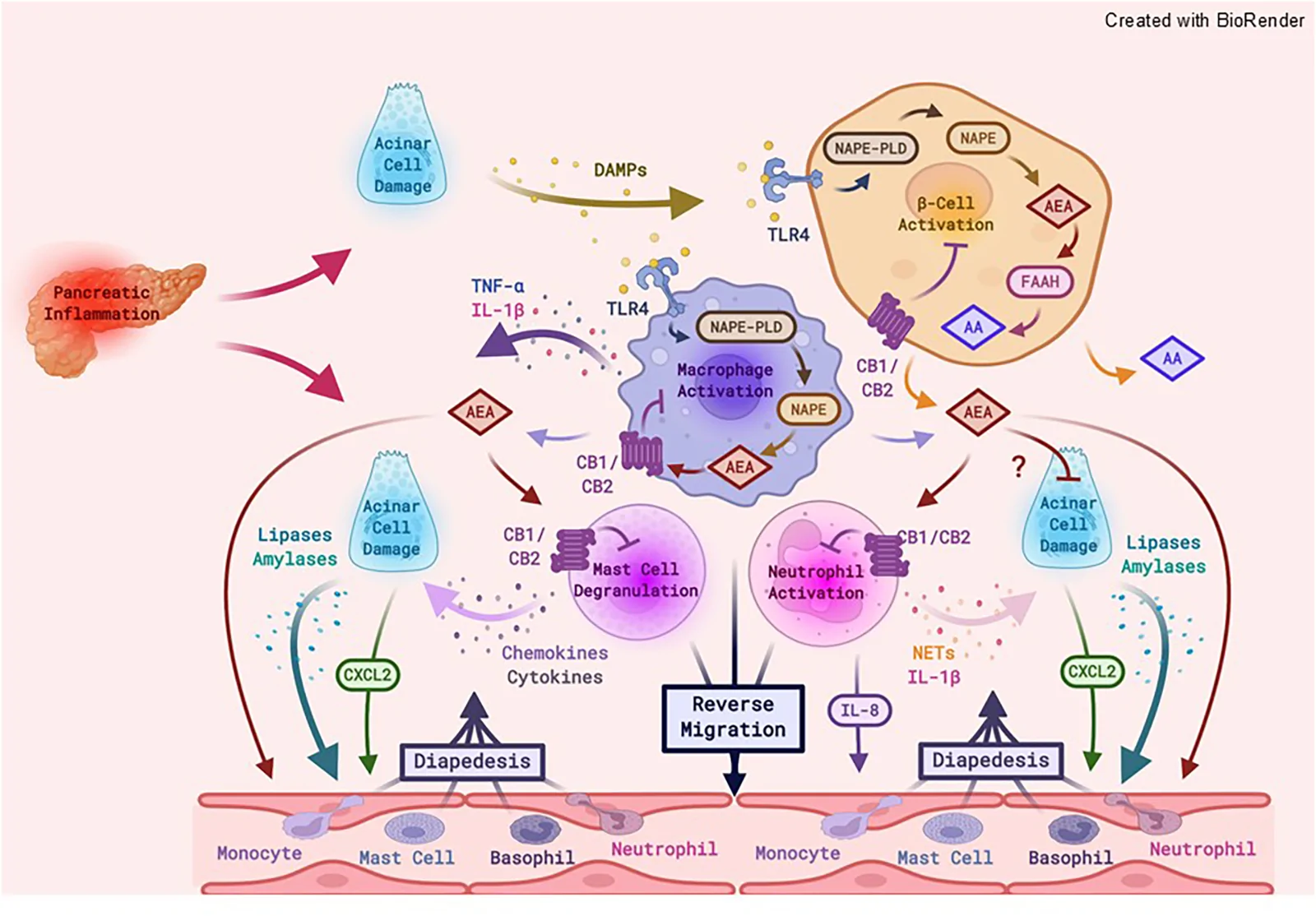
Proposed cellular framework of endocannabinoid-immune interactions during acute pancreatitis. Acinar cell injury initiates a local inflammatory response characterized by the release of damage-associated molecular patterns (DAMPs), activation of innate immune signaling pathways, and recruitment of circulating leukocytes. DAMP-mediated activation of Toll-like receptor 4 (TLR4) on pancreatic β-cells and macrophages promotes N-acyl phosphatidylethanolamine phospholipase D (NAPE-PLD)-dependent synthesis of the endocannabinoid anandamide (AEA) from membrane phospholipid precursors. Newly generated AEA acts on cannabinoid receptors expressed by immune cells to suppress inflammatory signaling and limit the release of pro-inflammatory cytokines, including tumor necrosis factor-α (TNF-α) and interleukin-1β (IL-1β). Endocannabinoid signaling also restrains activation of mast cells and neutrophils, thereby reducing the release of pro-inflammatory mediators, neutrophil extracellular traps (NETs), and digestive enzymes that contribute to acinar cell injury. Simultaneously, chemokines released by injured pancreatic tissue establish gradients that promote leukocyte recruitment from circulation.

In AP, transient activation of ECS pathways may therefore represent one component of a broader compensatory response that limits inflammation, suppresses pain, restrains PSC activation, and promotes tissue recovery (Cao et al., 2012; Dembiński et al., 2006; Michalski et al., 2007; Zhang et al., 2014). However, these effects have been demonstrated predominantly in rodent models and should not be assumed to occur identically in humans. By contrast, recurrent or persistent pancreatic injury may progressively impair this regulatory capacity. One proposed model (**Figure 3**) is that repeated injury could produce a state of relative endocannabinoid insufficiency, in which receptor expression and responsiveness remains altered while endogenous ligand availability becomes inadequate to sustain protective signaling. At present, however, this hypothesis has not yet been tested as a defining mechanism of chronic pancreatitis. If confirmed, such an imbalance could contribute to persistent inflammation, chronic abdominal pain, PSC activation, and progressive fibrogenesis, thereby promoting the transition from recurrent AP to CP (**Figure 3**). Longitudinal studies will be required to determine whether such changes occur consistently across the pancreatitis continuum and whether restoration of endogenous cannabinoid signaling can halt disease progression or reverse established pathology. Indeed, human pancreatic tissue studies indicate substantial remodeling of the ECS in CP, including reduced pancreatic concentrations of 2-AG together with increased CB_1_ and CB_2_ receptor expression in PSCs, intrapancreatic nerves, and inflammatory cells (Michalski et al., 2008). Because these observations are cross-sectional, they do not establish a causal deficiency state. Rather, the combination of reduced endocannabinoid ligand availability and increased receptor expression is consistent with a proposed state of relative endocannabinoid insufficiency, a hypothesis that will require longitudinal and functional validation. Separately, *in vitro* experiments using isolated PSCs have shown that pharmacological cannabinoid receptor activation can suppress PSC activation and reduce the expression of fibrogenic and inflammatory markers, supporting a direct modulatory effect of cannabinoid signaling on PSC phenotype. These findings provide a rationale for the proposed framework but do not establish that endocannabinoid insufficiency drives CP progression *in vivo*.

**Figure 4** presents a proposed cellular framework through which ECS signaling may coordinate inflammatory and pain responses within the injured pancreas. Multiple pancreatic and extra-pancreatic cell populations—including acinar cells, endocrine cells, immune cells, PSCs, and sensory neurons—express components of the ECS and are therefore positioned to participate in local endocannabinoid communication (Li et al., 2010, 2012; Starowicz et al., 2008). Following pancreatic injury, endocannabinoid release may regulate immune activation, sensory neuron excitability, and fibrogenic responses through cannabinoid receptors and related lipid-signaling pathways (Michalski et al., 2007; Zhang et al., 2014). Although the specific cellular origin of endocannabinoids and the relative contribution of individual signaling pathways remain to be determined, current evidence indicates that ECS signaling represents an important neuroimmune regulatory network within the pancreas. However, not all interactions depicted in **Figure 4** have been directly demonstrated within the same pancreatitis model, and the figure should therefore be viewed as a conceptual framework for future mechanistic testing.

From a translational perspective, ECS-targeted approaches are particularly attractive because they may provide opportunities to address two major unmet clinical challenges in pancreatitis: disease modification and pain management. Unlike conventional analgesic approaches that primarily target symptoms, ECS-directed interventions could potentially influence inflammation, fibrosis, immune activation, and nociceptive processing simultaneously. However, therapeutic outcomes are likely to depend on the receptor or metabolic pathway targeted, the cellular compartment involved, the cannabinoid composition used, and the stage and severity of pancreatic disease.

Despite encouraging preclinical findings, evidence supporting endocannabinoid-based therapies in individuals with AP remains largely observational and conflicting. Case reports and systematic reviews have implicated cannabis as a potential cause of AP, particularly among younger individuals and frequent users (Barkin et al., 2017; Grant and Gandhi, 2004; Lubega and Lwanga, 2021; Tanvir et al., 2024). Recurrence with continued cannabis exposure and resolution following cessation in some cases provide temporal support for a possible association, although these observations cannot establish causality and may be confounded by incompletely assessed alternative etiologies. Conversely, large retrospective database studies have associated cannabis use with reduced inpatient mortality, shorter hospital stays, lower healthcare costs, and fewer severe complications among patients hospitalized with AP (Simons-Linares et al., 2019). Cannabis use has also been associated with less severe alcohol-associated pancreatitis and a lower risk of AP among individuals with alcohol use disorder, whereas similar associations have not been observed in gallstone-associated disease (Adejumo et al., 2019; Goyal et al., 2017). Together, these findings suggest that patient characteristics and pancreatitis etiology may substantially influence the observed relationship between cannabis exposure and clinical outcomes. Thus, the apparent discrepancies across studies may reflect heterogeneity in cannabinoid exposure and patient populations rather than a simple protective or harmful effect of cannabis.

Clinical evidence in CP is similarly limited and heterogeneous. In a randomized crossover trial involving patients with CP-associated abdominal pain, a single oral dose of Δ^9^-THC (Namisol^®^) did not provide significant analgesia compared with active placebo and was associated with increased heart rate and anxiety (de Vries et al., 2016). Δ^9^-THC also failed to significantly alter circulating TNF-α, IL-8, or IL-10 concentrations (Utomo et al., 2015). Although this trial provides stronger evidence than observational studies, its interpretation is limited by the small sample size, single-dose design, and use of a defined Δ^9^-THC formulation rather than prolonged treatment with other cannabinoid preparations. Whether different doses, formulations, routes of administration, or treatment durations would yield different outcomes therefore remains uncertain. By contrast, retrospective studies of medical cannabis use have reported reductions in opioid consumption, hospital admissions, and emergency-department utilization in patients with painful CP, while larger database analyses have associated cannabis use with lower mortality and fewer complications (Barlowe et al., 2019; Sohal et al., 2025). However, these studies lack the pharmacological control of randomized trials, and cannabis exposure may vary substantially in Δ^9^-THC and CBD content, dose, formulation, route, frequency, and duration. In addition, treatment selection may be influenced by disease stage, pain severity, prior opioid exposure, healthcare access, comorbidities, and patient preference. These factors introduce substantial potential for confounding and make direct comparison with controlled Δ^9^-THC administration difficult.

### 5.1 Future directions

The need for improved treatments for pancreatic pain was highlighted by the 2021 National Institute of Diabetes and Digestive and Kidney Diseases (NIDDK) workshop, which identified pancreatic pain as a major research priority and emphasized substantial gaps in mechanistic understanding and therapeutic options (Uc et al., 2021). Among the priorities proposed was a better understanding of the contribution of endocannabinoid signaling and the therapeutic potential of ECS-targeted interventions in pancreatic pain.

Future studies should define the cellular and temporal remodeling of ECS signaling during pancreatic injury. Single-cell and spatial transcriptomics, targeted and untargeted lipidomics, advanced imaging, and cell type-specific genetic models could help identify which pancreatic and immune-cell populations produce or respond to endocannabinoids at different stages of disease. Longitudinal studies will be particularly important because most existing investigations examine isolated time points, making it difficult to determine whether ECS alterations are protective, compensatory, maladaptive, or secondary to tissue injury. Tracking endocannabinoid concentrations, receptor expression, enzyme activity, immune-cell phenotypes, pain behaviors, and fibrosis across the progression from AP to recurrent AP and ultimately CP will be necessary to establish temporal and causal relationships and to directly test the proposed adaptive or resilience framework and the possibility of relative endocannabinoid insufficiency.

From a clinical perspective, adequately powered randomized controlled trials will be needed to determine whether cannabis-derived compounds, selective cannabinoid receptor ligands, or pharmacological approaches that enhance endogenous cannabinoid signaling provide meaningful benefit in patients with pancreatitis. These trials should use chemically defined formulations with standardized doses, routes of administration, and treatment durations, while prospectively characterizing patients according to pancreatitis etiology, disease stage, pain phenotype, prior and concomitant opioid exposure, relevant comorbidities, and previous cannabis exposure. Such stratification may help identify patient subgroups most likely to benefit from specific ECS-targeted interventions. Future studies should also distinguish disease-modifying effects from symptomatic analgesia by incorporating outcomes beyond pain intensity, including opioid consumption, hospitalizations, functional status, quality of life, central sensitization, inflammatory and metabolic biomarkers, and progression from recurrent AP to established CP. This broader approach will be essential to determine whether ECS-directed therapies merely relieve symptoms or can modify the underlying disease process.

Another important research direction should examine the broader endocannabinoid-related signaling network, including noncanonical targets such as PPARα, TRPV1, and GPR55, as well as the endogenous lipid mediators PEA and OEA. These pathways are pharmacologically distinct from classical cannabinoid receptors and may act independently while also functionally converging with cannabinoid signaling, including in the regulation of pain and inflammation (Piomelli and Mabou Tagne, 2022). Recent human evidence also supports a potential role for paracannabinoid signaling in pancreatic disease. Individuals reporting abdominal pain exhibited lower circulating concentrations of 2-AG, OEA, and PEA, while pain severity was inversely correlated with circulating 2-AG and OEA levels (Goodman et al., 2025). Whether and how these pathways interact within specific pancreatic cell populations during AP or CP remains largely unknown. Defining these relationships may reveal therapeutic strategies that preserve beneficial endocannabinoid-related signaling while minimizing adverse effects associated with widespread cannabinoid receptor activation.

### 5.2 Conclusions

The ECS represents a promising yet complex therapeutic landscape in pancreatic disease. Current evidence supports an important role for endocannabinoid-related signaling in the regulation of inflammation, nociception, immune responses, and fibrogenesis, but many of the underlying mechanisms remain incompletely defined. Despite encouraging preclinical data, human evidence supporting cannabinoid-based interventions in AP and CP remains limited and inconsistent, suggesting that ECS actions are highly context-dependent, and may shift from protective to harmful depending on cell type, disease stage, and the specific signaling pathways engaged. Resolving these complexities will require coordinated mechanistic, longitudinal, and translational studies to clarify the cellular sources, temporal dynamics, and functional consequences of ECS signaling. Such efforts may provide a more precise understanding of when, where, and in whom ECS signaling is beneficial or maladaptive, and could ultimately enable the development of pathway-specific and patient-tailored therapeutic strategies that alleviate pancreatic pain, limit inflammatory and fibrotic progression, and improve outcomes in patients with acute and chronic pancreatitis.
